# A Rare Case of Recurrent Parathyroid Adenomas After Initial Parathyroidectomy

**DOI:** 10.7759/cureus.44849

**Published:** 2023-09-07

**Authors:** Grant N Schalet, Luke Vincent, Carl Eguez, Gerardo Diaz, Mark S Shachner

**Affiliations:** 1 Surgery, Broward Health Medical Center, Fort Lauderdale, USA; 2 Surgery, Nova Southeastern University Dr. Kiran C. Patel College of Osteopathic Medicine, Fort Lauderdale, USA; 3 Surgery, Broward Health Coral Springs, Coral Springs, USA

**Keywords:** parathyroidectomy, persistent primary hyperparathyroidism, parathyroid neoplasms, parathyroid gland adenoma, adult primary hyperparathyroidism

## Abstract

Hyperparathyroidism usually presents asymptomatically with elevated levels of calcium and parathyroid hormone; this biochemical imbalance establishes the diagnosis. In 80-85% of cases of primary hyperparathyroidism, singular parathyroid adenomas occur. In rare cases, this problem occurs due to multiple adenomas, multiglandular hyperplasia, or parathyroid carcinoma. Recurrent primary hyperparathyroidism (R-PHPT), as demonstrated in this case, is defined as hypercalcemia that arises after six months of normocalcemia following initial surgery for PHPT. The aim of this report is to describe the diagnosis and management of three parathyroid adenomas in a patient, two of which occurred after an initial partial parathyroidectomy.

## Introduction

Parathyroid adenomas are benign tumors that are responsible for the vast majority of primary hyperparathyroidism (PHPT). The prevalence of this common endocrine disease is 0.1-0.3% of the population and is more likely to occur in women [1]. The common presentation consists of elevated levels of calcium and parathyroid hormone (PTH); this biochemical imbalance establishes the diagnosis despite the fact that the patients are often asymptomatic. Primary hyperparathyroidism occurs as a singular parathyroid adenoma in 80-85% of cases. Rarely it can arise from multiple adenomas or multiglandular hyperplasia (5-10%) or a parathyroid carcinoma (<1%) [2]. Common symptoms of symptomatic primary hyperparathyroidism are related to hypercalcemia and hypercalciuria, including fatigue, weakness, abdominal pain, kidney stones, excessive urination, and psychiatric disturbances [3]. Typically, a targeted dissection can be performed when the disease is localized with preoperative imaging. However, with recurrent PHPT or multiple adenomas, bilateral neck exploration is usually necessary. Localization and treatment success has improved with more advanced imaging. In this case report, we share a rare case of double adenoma recurrence 12.5 years after a primary adenoma was resected.

## Case presentation

This case report describes a 64-year-old woman with recurrent primary hyperparathyroidism who underwent a focused right parathyroidectomy in 2008. Initially, the patient was referred to the surgery team due to osteoporosis on a dual-energy x-ray absorptiometry (DEXA) scan, hypercalcemia of 11.8 mg/dL (reference range 8.4-10.2 mg/dL), and a parathyroid hormone (PTH) level of 65.7 mg/mL (reference range 8.5-72.5 mg/mL). The patient denied depression, confusion, and constipation and did not have a history of kidney stones or renal insufficiency. Her past medical history included anxiety, gastritis, gastroesophageal reflux disease, hypercholesterolemia, and irritable bowel syndrome. She had a family history of arthritis and cataracts from her mother and kidney stones from her father and brother, but no family history of known hyperparathyroidism. Pertinent medications included cholecalciferol (vitamin D3) 1,250 mcg (50,000 IU) daily. She had also, notably, undergone thyroid ablation years prior. 

Prior to her index operation, a sestamibi scan was performed, which showed persistent uptake in the right lower aspect of the thyroid gland. These findings were concerning for a single parathyroid adenoma of the right inferior gland. The patient underwent a focused parathyroidectomy of the right inferior lobe with the utilization of intraoperative PTH monitoring. She had a baseline PTH of 65 mg/mL and a pre-excision PTH of approximately 75 mg/mL. The 5-minute post-excision PTH dropped to 19.5 mg/mL and the 10-minute post-excision PTH dropped to 12.6 mg/mL, both representing greater than a 50% drop from baseline. Her pathology revealed "proliferation of predominantly water-clear cells having a vaguely lobar configuration" consistent with parathyroid adenoma. Her post-operative PTH and calcium levels during outpatient follow-up were also within normal limits. 

**Figure 1:**
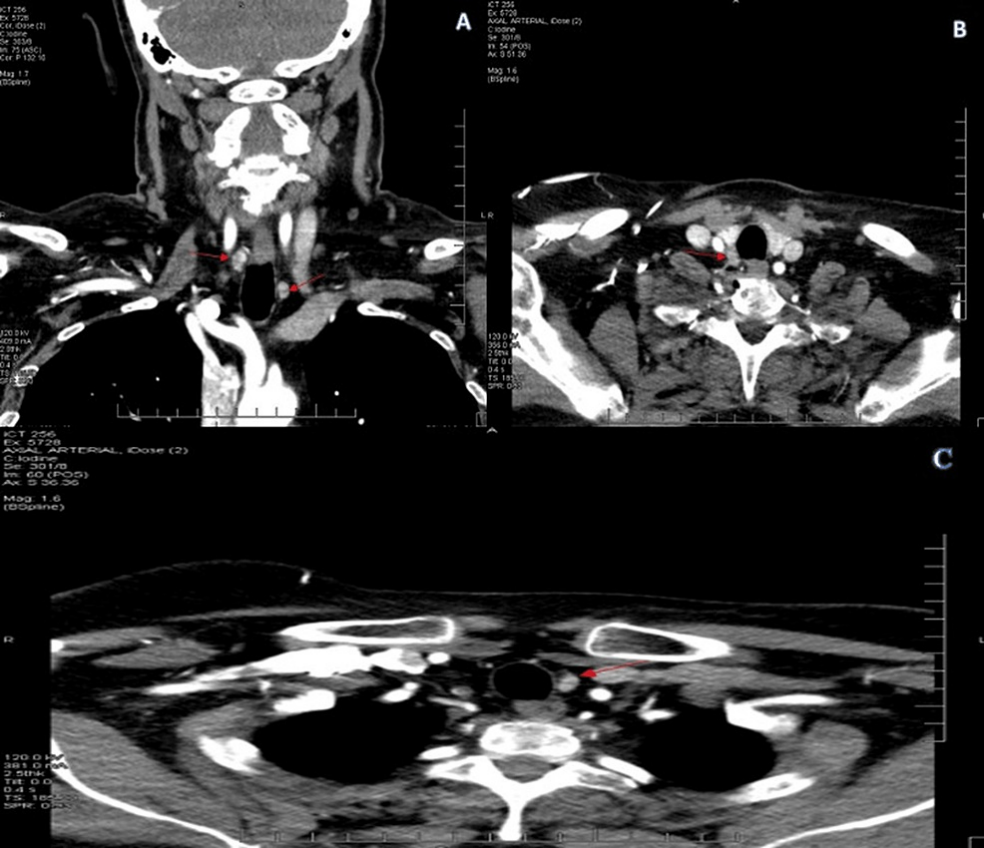
Four-dimensional computed tomography imaging was obtained prior to the patient’s bilateral neck exploration, demonstrating evidence of both the enlarged left inferior parathyroid nodule and right superior parathyroid nodule (red arrows). Panel A: Coronal view demonstrating the bilaterally enlarged parathyroid nodules (red arrows). Panel B: Axial view demonstrating the right-sided enlarged parathyroid nodule (red arrow). Panel C: Axial view demonstrating the left-sided enlarged parathyroid nodule (red arrow).

Despite demonstrating post-operative normalization of her parathyroid hormone (PTH) and calcium levels and resolution of her symptoms, she required secondary surgery twelve years later for recurrent adenomas. On routine outpatient lab work, she was noted to have hypercalcemia at 11.3 mg/dL (reference range 8.6-10.4 mg/dL), and she was again referred to the surgeon for evaluation. Repeat labs were obtained, recording a calcium of 11.4 mg/dL (reference range 8.6-10.4mg/dL), ionized calcium of 6.3 mg/dL (reference range 4.8-5.6mg/dL), and parathyroid hormone of 71 mg/dL (reference range 14-64 mg/dL). The patient underwent a sestamibi scan, which was negative. However, a four-dimensional computed tomography (4-D CT) scan of the neck with and without intravenous contrast demonstrated an enlarged left inferior parathyroid nodule measuring 10 × 8 mm and a right superior parathyroid nodule measuring 13 × 10 mm consistent with bilateral enlarged parathyroid glands (Figures 1A-1C).

During the second operation, a bilateral neck exploration with the evaluation of the three remaining parathyroid glands was performed; cranial nerve electromyography (EMG) monitoring and intraoperative Neoprobe measurements were also utilized. Background measurements with the Neoprobe using a collimator were 380 counts per second (cps). The left inferior parathyroid gland had a firm nodule and was excised. The Neoprobe measurement of this gland measured 100 cps, which was almost 30% of background uptake consistent with an adenoma. The left upper parathyroid gland was noted to be in a normal position and of normal size and consistency. Frozen section biopsy was performed on this gland, leaving it otherwise intact. It was noted to have 12-15 counts, which is less than 4% of the background. The right upper parathyroid gland was noted to be firm and enlarged. This gland was excised, and a Neoprobe measurement returned with 65 cps, which is 17% of the background. Since the patient’s right inferior parathyroid gland was removed in the 2008 index operation, no further dissection was performed. The right upper and left lower parathyroid glands were excised, and the left upper gland was biopsied.

**Figure 2:**
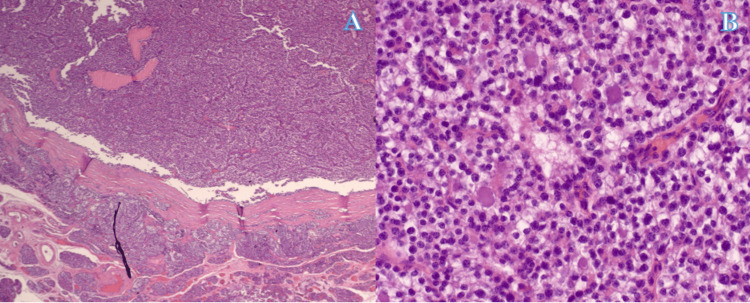
Hypercellular parathyroid tissue on histological slides using hematoxylin and eosin (H&E) staining obtained from the surgical specimen, consistent with parathyroid adenoma. The normal rim of parathyroid tissue is shown along the bottom half of panel A (40× magnification), juxtaposed with the hypercellular tissue at the top half of panel A and throughout panel B (200× magnification).

Within 24 hours after the procedure, the patient’s serum calcium was 8.9 mg/dL. The final pathology report revealed left lower parathyroid hypercellular tissue consistent with parathyroid adenoma and right superior parathyroid tissue with fibrosis consistent with parathyroid adenoma as well as left upper parathyroid tissue with no significant pathologic diagnosis (Figures 2A, 2B).

Postoperatively, she exhibited no signs or symptoms of hypocalcemia. On post-operative day one, the patient was afebrile, reported feeling well with no pain, and was deemed safe for discharge.

## Discussion

The aim of this report is to describe a patient in whom two new adenomas arose, 12.5 years after a single adenoma was resected. This patient had a long intervening period of normocalcemia and normal PTH levels. Primary hyperparathyroidism can be identified by laboratory studies alone. A concurrent elevation in calcium and PTH is the emblem of primary hyperparathyroidism. Fine needle aspiration may be used for histopathology, but this method of diagnosis cannot distinguish correctly between different types of parathyroid pathologies [4,5]. No imaging modalities are required to establish the diagnosis, but they are helpful in preoperative planning. Image-guided localization of the lesion helps to decrease perioperative complications and time spent in the operating room [6]. Non-invasive imaging modalities include parathyroid scintigraphy using technetium (^99m^Tc) sestamibi, ultrasound (US), 4D-CT, and magnetic resonance imaging (MRI). 

Preoperative localization is mostly done by ultrasound guidance or a sestamibi scan. If these modalities fail, then a CT-4D scan and/or four-gland neck exploration, as demonstrated in Figures 1, 2 are indicated. For our patient with a negative sestamibi scan before the second operation, a 4D-CT had been ordered by the referring physician. 4D-CT utilizes multiplanar images and perfusion characteristics to identify abnormal parathyroid glands. In a study by Lubitz et al., of 60 patients, 4D-CT correctly lateralized 73% and localized 60% of abnormal glands found at operation with an accuracy of 70% [7]. 4D-CT identifies more than half of abnormal parathyroids missed by traditional imaging and should be considered in cases with negative or discordant sestamibi and ultrasound [7]. 

The literature has demonstrated that patients may have two or more hyperfunctioning glands despite the fact that pre-operative imaging indicates single-gland disease [8]. According to the American Association of Endocrine Surgeons (AAES), when performing focused parathyroidectomy, intraoperative PTH monitoring should be employed as an adjunct to pre-operative imaging modalities, citing cure rates between 97 and 99% [9]. In doing so, the risk of missed double-gland or multi-gland disease is minimized. Although the authors suspect that recurrent adenomas are the etiology of this patient's hypercalcemia following the index operation, a missed adenoma is another less likely possibility.

During the index operation, intra-operative PTH level monitoring was performed. By measuring PTH levels prior to excision, 5 minutes after excision, 10 minutes after excision and by checking for a greater than 50% decrease in PTH from baseline and from pre-excision, the risk of leaving behind hyper-functioning parathyroid tissue was minimized. Multiple criteria exist that incorporate these values to assess the adequacy of resection. The PTH levels measured from the index case satisfied both the Miami and Vienna criteria, which provide surgeons with a quantitative threshold for concluding (or continuing) the operation after removing a suspicious parathyroid lesion. Although other criteria exist, the Miami and Vienna criteria have the highest overall accuracy, at 97.3% and 92.3%, respectively [9]. 

Radio-guided parathyroidectomy was utilized in the second operation in order to obtain a real-time evaluation of the hormonal activity within each gland, avoiding the need to obtain multiple frozen sections and the need for intraoperative PTH testing. Additionally, all three remaining glands were explored. With the use of the Neoprobe, our patient demonstrated abnormal uptake in the two abnormally appearing parathyroid glands and a normal gland with the expected low radioactivity. Norman and Politz describe outstanding post-operative outcomes with radio-guided parathyroidectomy, citing 5,000 operations during which a gamma probe was used to instantaneously measure parathyroid gland radioactivity. Greater efficiency with instantaneous feedback about the gland’s physiologic activity enabled the authors to determine whether a patient had a hypersecretory adenoma that warranted resection [10]. This approach was successfully utilized in this case.

The patient’s history of prior radiation exposure from her thyroid ablation could have also increased her risk of developing recurrent hyperparathyroidism. In the literature, prior radiation exposure has proven to lead to a generally increased prevalence of hyperparathyroidism [11]. This was proven in a study focused on the long-term sequelae of radiation exposure from the atomic bombings in Hiroshima and Nagasaki. In a 2020 paper by Parikh et al., they note that patients with prior neck radiation do have a greater risk for the development of a second adenoma in a previously normal gland [12]. Although our patient’s recurrence may not necessarily have a direct connection with her prior radioactive thyroid ablation, we can certainly draw the conclusion that she was at an increased risk of developing her recurrent disease when we take into consideration her prior radiation exposure.

## Conclusions

Multiglandular disease does occur in approximately 10-15% of patients at the index surgery, and this case demonstrates the management of a rare multi-glandular recurrence. In addition, the authors provide an efficient, effective approach to managing recurrent adenomatous disease. Radio-guided parathyroid exploration is recommended to identify and resect hypersecretory glands. This intervention will enable patients to experience physiologic normalization of their PTH and calcium levels after surgery and in the years thereafter. 
